# An Interpretable System for Screening the Severity Level of Retinopathy in Premature Infants Using Deep Learning

**DOI:** 10.3390/bioengineering11080792

**Published:** 2024-08-05

**Authors:** Wenhan Yang, Hao Zhou, Yun Zhang, Limei Sun, Li Huang, Songshan Li, Xiaoling Luo, Yili Jin, Wei Sun, Wenjia Yan, Jing Li, Jianxiang Deng, Zhi Xie, Yao He, Xiaoyan Ding

**Affiliations:** 1 State Key Laboratory of Ophthalmology, Guangdong Provincial Key Laboratory of Ophthalmology and Visual Science, Zhongshan Ophthalmic Center, Sun Yat-sen University, Guangzhou 510060, Chinaxiezhi@gmail.com (Z.X.); 2 Department of Ophthalmology, Guangdong Eye Institute, Guangdong Provincial People’s Hospital (Guangdong Academy of Medical Sciences), Southern Medical University, Guangzhou 510080, China; 3 Department of Ophthalmology, Guangdong Women and Children Hospital, Guangzhou 511400, China

**Keywords:** deep learning, retinopathy of prematurity (ROP), homologous pre-training, domain adaptation

## Abstract

Accurate evaluation of retinopathy of prematurity (ROP) severity is vital for screening and proper treatment. Current deep-learning-based automated AI systems for assessing ROP severity do not follow clinical guidelines and are opaque. The aim of this study is to develop an interpretable AI system by mimicking the clinical screening process to determine ROP severity level. A total of 6100 RetCam Ⅲ wide-field digital retinal images were collected from Guangdong Women and Children Hospital at Panyu (PY) and Zhongshan Ophthalmic Center (ZOC). A total of 3330 images of 520 pediatric patients from PY were annotated to train an object detection model to detect lesion type and location. A total of 2770 images of 81 pediatric patients from ZOC were annotated for stage, zone, and the presence of plus disease. Integrating stage, zone, and the presence of plus disease according to clinical guidelines yields ROP severity such that an interpretable AI system was developed to provide the stage from the lesion type, the zone from the lesion location, and the presence of plus disease from a plus disease classification model. The ROP severity was calculated accordingly and compared with the assessment of a human expert. Our method achieved an area under the curve (AUC) of 0.95 (95% confidence interval [CI] 0.90–0.98) in assessing the severity level of ROP. Compared with clinical doctors, our method achieved the highest F1 score value of 0.76 in assessing the severity level of ROP. In conclusion, we developed an interpretable AI system for assessing the severity level of ROP that shows significant potential for use in clinical practice for ROP severity level screening.

## 1. Introduction

Retinopathy of prematurity (ROP) is the most widely recognized cause of visual impairment after pre-term birth, with almost 220,000 prevalent cases of blindness and vision loss due to ROP worldwide [1]. Early screening can reduce child blindness and the relative cost of treatment. However, the lack of cost-effective screening strategies that can feasibly be implemented in clinical practice has led to the current global cost of ROP screening remaining high [2,3,4,5,6].

The early treatment for retinopathy of prematurity (ETROP) study guidelines [7] define severe ROP, which requires treatment, as a combination of three components: staging, zoning, and plus disease. According to the international classification of retinopathy of prematurity (ICROP3) [8], staging is determined by the type of the most severe ROP lesion (five stages), zoning is determined by the distance from the most severe lesion to the optic disc (three zones), and plus disease is defined as the dilation and tortuosity of retinal vessels within zone I. 

Current ROP screening relies on skilled ophthalmologists performing binocular indirect ophthalmoscope exams or interpreting wide-field digital retinal imaging (WFDRI), requiring substantial ophthalmologist time and effort. However, experienced ophthalmologists are limited. AI solutions have been suggested to reduce this workload burden [9,10]. Existing AI research on ROP severity level screening mainly contains two categories. One category involves the annotating of data with severity level of ROP by experienced doctors and then utilizing a classification model to predict the final severity level [11,12,13,14,15,16,17,18,19,20,21,22,23,24]. The annotations can be fully or partially based on the clinical guideline; for example, some studies incorporate stage as the severity factor since it helps determine treatment needs [11,14,15,16,17,21]. The other category uses similar annotations but utilizes multi-modal data, for example, RetCam Ⅲ images and clinical reports, to provide age, birth weights [25], and time series oxygen data [26] to predict the severity level.

AI medical models for retinopathy of prematurity (ROP) screening must prioritize transparency and interpretability in their diagnostic processes [27,28,29], yet current AI studies often fail to meet these standards. Both direct prediction of ROP severity level or many studies on staging [30,31,32,33,34,35], zoning [36,37,38,39,40], and plus disease [33,41,42,43,44,45,46,47,48,49] predictions utilize black-box CNN models, which inherently lack transparency. While combining these models to mimic clinical processes is feasible, it still falls short in providing necessary clarity. This opacity hinders clinicians in assessing diagnostic evidence, contradicting evidence-based medicine principles and undermining trust in AI results [50,51]. Moreover, the inability to communicate clear explanations to patients leads to lost clinical information and reduced satisfaction [52]. Therefore, developing interpretable AI that aligns with clinical guidelines is crucial for fostering trust, enabling result verification, and promoting patient-centered care in ROP screening and broader medical diagnostics.

Our method significantly enhances clinical interpretability in ROP screening by mapping lesions from individual images onto a panoramic view with zoning templates, simultaneously presenting staging and zoning information. This transparent approach aligns closely with the clinical diagnostic process, boosting doctors’ confidence in the results. Unlike previous studies, our model not only identifies but also locates individual retinal lesions, incorporating key diagnostic criteria—stage, zone, and plus disease presence—directly from images, which is a first in adhering to clinical screening protocols. The model’s visually interpretable decisions enable interactive reviews by ophthalmologists, reducing misdiagnosis risks. Comparative testing on external datasets establishes great performance improvement over ophthalmologists’ review. By leveraging domain adaptation to close the domain gap, our model achieves high accuracy for ROP assessment from WFDRI images. This feasibility for real-world severity screening promises to enhance timely treatment and reduce vision impairment for premature infants.

## 2. Methods

### 2.1. DATA Preparation and Preprocessing

The data in this study were collected from two hospitals in China: Guangdong Women and Children Hospital at Panyu (PY) and Sun Yat-sen University-affiliated Zhongshan Ophthalmic Center (ZOC). The PY dataset, collected from January 2015 to March 2018, was divided into training and validation subsets for lesion detection tasks. The ZOC dataset, collected from April 2018 to March 2022, served as an independent external validation dataset for the severity levels of ROP screening.

The images were captured directly with a contact retinal camera (RetCam Ⅲ, Clarity Medical System, 5775 W. Las Positas Blvd. Pleasanton, CA 94588 USA) and were exported directly from the device in PNG and JPG formats. Patient data collection and anonymization were carried out at each contributing center. Ethical approval for research use was obtained from each center (The ethics committee of Zhongshan Ophthalmic Center, Sun Yat-sen University (2020KYPJ175); the ethics committee of Guangdong Women and Children Hospital (202201057)). 

There are 520 pediatric patients, totaling 3330 images with ROP disease from PY. For the PY dataset, the original resolution is 640 × 480. We split the PY dataset into 80% for lesion detection model training and 20% for validation (Table 1). For the ZOC dataset, the original resolution is 640 × 480 or 600 × 1200 (resized to 640 × 480). There are 81 pediatric patients, totaling 136 eyes, 2770 images of ROP disease for system test, and 9,275,644 images from 9546 patients’ unlabeled images for pre-training and domain adaptation. 

Two experienced ophthalmologists independently annotated the datasets, which were reviewed by a clinical professor and served as the reference standard diagnosis. In case of disagreements, the majority opinion was considered. The PY dataset annotated the type and location of the lesions. The ZOC dataset annotated the stage, zone, and the presence of plus disease in ROP.

Five ophthalmologists, including two senior doctors with around six years of experience and three junior doctors with around two years of experience, were invited to read the ZOC datasets, including the stage, zone, and the presence of plus diseases in ROP.

### 2.2. Disease Classification Criteria

According to ETROP study guidelines [7], we classify all lesions in zone Ⅰ, stage Ⅱ lesions with plus lesions in zone Ⅱ, stage Ⅲ lesions in zone Ⅱ, and stage Ⅳ lesions as high risk of severe ROP. There are no stage Ⅴ lesions here; stage Ⅴ lesions are inherently rare, and once stage Ⅴ lesions are discovered, they are promptly treated. Lesions in other categories are considered mild. As many as 107 eyes from 64 pediatric patients are mild and 29 eyes from 24 pediatric patients are severe in the ZOC dataset.

### 2.3. The Interpretable ROP Assessment System

**Figure 1:**
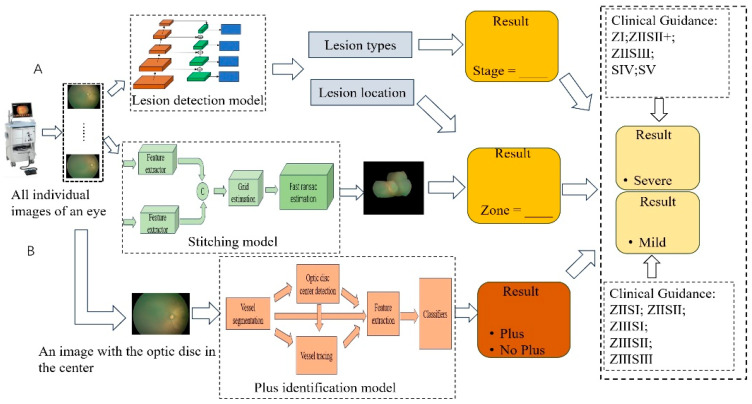
The workflow for automatic assessment of the severity level of retinopathy of prematurity: (**A**) represents the data collection, model training and prediction, and lesion stitching, and finally predicts the stage and zone results of ROP; (**B**) represents data collection, predicting plus disease and obtaining the final result of whether each eye has plus disease or not. The severity grade is ultimately inferred based on the stage, zone, and whether it is a plus lesion in ROP according to clinical guidelines. Z I represents zone I; Z II S II+ represents zone II and stage II with plus disease.

Architecture: The assessment system includes the following steps (Figure 1): (1) All orientation images from one eye are input into the lesion detection model. (2) The lesion detection model detects the type and location of lesions from each image; the stage result is calculated from the lesion type. (3) All orientation images from one eye are combined via the stitching model [53] into a panoramic view for each eye. (4) The zone result is determined by combining the panoramic view and location of lesions. (5) An image with the optic disc in the center is input into the plus identification model, adapted from I-ROP ASSIST [46] to predict the presence of the plus disease. (6) The severity grade of each eye is inferred based on the stage, zone, and the presence of plus disease according to clinical guidelines.

The process of obtaining interpretable results: First, the lesion detection model predicts the types and locations of potential lesions. Subsequently, this information is mapped onto panoramic images of each eye. Finally, panoramic images of individual eyes with staging and zoning information are formed.

**Figure 2:**
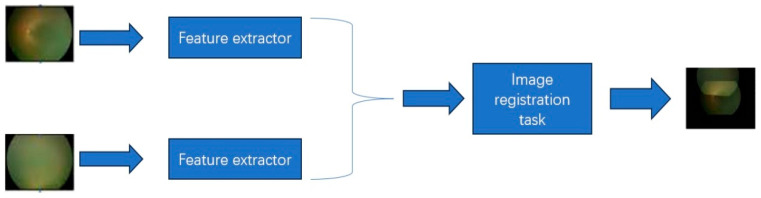
The flowchart of pre-training [53]. Two retinal images were sent to a feature extraction module based on ResNet50 and an image registration prediction module, resulting in a registered image. The model weights generated during this process were used for downstream task training.

**Figure 3:**
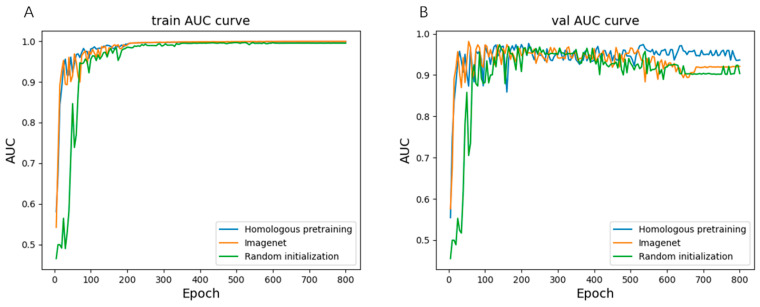
The performance of three training strategies for lesion detection: (**A**) represents the AUC metric for the training set; (**B**) represents the AUC metric for the validation dataset.

Development of the lesion detection model: The lesion detection model was developed based on the RetinaNet framework [54], a classical framework in the field of object detection, and Resnet 50 backbone [55]. We used the homogeneous pre-training to train the backbone via self-supervised learning via the registration of images from the same orientation using images from the unlabeled ZOC dataset. Data enhancements were performed, including random flip, gaussian blur, green channel random, CLAHE random, photo metric distortion, shear, and rotate. The workflow of the pretrain model is illustrated in Figure 2; the network architecture used for feature extraction is based on ResNet50. The image registration task of the pre-trained model involves leveraging the extracted features to predict registration parameters. Pytorch deep learning framework and a V100 GPU were used to train the model. The loss function used is focal loss [56], with the Adam optimizer. The initial learning rate was set at 0.0001. The batch size is set at 8, with the epoch of 800. The performance of random initialization and ImageNet transfer learning and our proposed homogeneous pre-training for lesion detection is shown in Figure 3.

**Figure 4:**
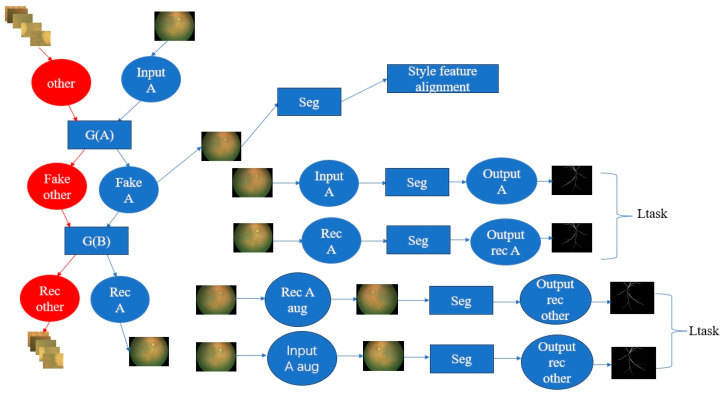
The flowchart of domain adaptation. The target domain ZOC images and their cropped patches are transformed into the flowchart of domain adaptation based on CycleGAN. The blue parts in the image represent the processing module or results for the entire fundus image in the ZOC, while the red parts represent the processing module or results for the cropped patches from ZOC. We utilize the source domain PY vessel segmentation task model and the feature style alignment module to constrain the model. The final output will be images with a style similar to the source domain PY data.

Domain adaptation: The lesion detection model and the plus identification model were trained using a PY dataset domain, which is different from the ZOC dataset such that domain adaptation was performed on the ZOC unlabeled dataset by transforming it into the PY dataset domain using the cycle-GAN framework [57], enhancing the generalization performance of our model. The workflow of domain adaptation is illustrated in Figure 4.

### 2.4. Evaluation Metrics

The kappa (1) and accuracy were used to evaluate the performance of stage and zone of ROP. The higher the value of kappa, the better the performance. The accuracy and F1 were used to evaluate the performance of plus disease classification. The accuracy (2), sensitivity (3), specificity (4), and F1-score (5), as well as area under the curve (AUC), were used to evaluate the performance of the severity levels of ROP.

(1) $$kappa=\frac{p_{0}-p_{e}}{1-p_{e}}$$

(2) $$accuracy=\frac{TP+TN}{TP+TN+FP+FN}$$

(3) $$sensitivity=\frac{TP}{TP+FN}$$

(4) $$specificity=\frac{TN}{TN+FP}$$

(5) $$F1=\frac{2*precision*sensitivity}{precision+sensitivity}$$

### 2.5. Experiments Setting

To verify whether homologous pre-training models and domain adaptation techniques contribute to improving the level of severity assessment in downstream ROP, we conducted comparative experiments. We designated the use of homologous pre-training and domain adaptation techniques as method 1, random initialization and domain adaptation as method 2, ImageNet and domain adaptation as method 3, homologous pre-training as method 4, random initialization as method 5, ImageNet as method 6.

## 3. Results

### 3.1. Evaluation of the Performance for Classifying the Stage of ROP

**Figure 5:**
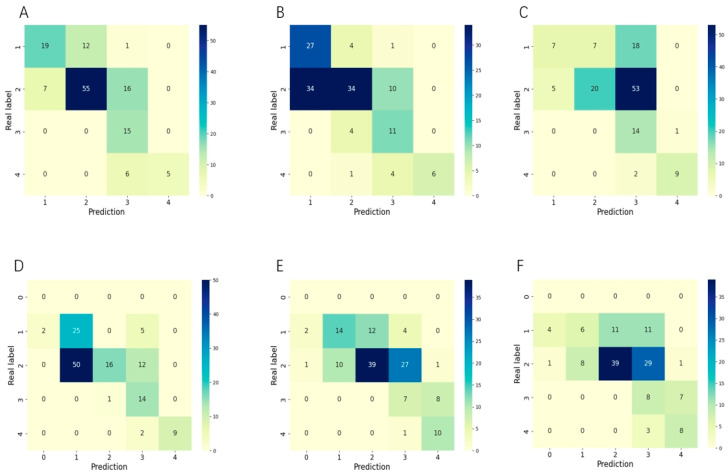
The confusion matrices of our method and clinical doctors in assessing the stage of ROP tasks: (**A**) represents our system; (**B**) represents clinical doctor A; (**C**) represents clinical doctor B; (**D**) represents clinical doctor X; (**E**) represents clinical doctor Y; (**F**) represents clinical doctor Z. The horizontal axis of the confusion matrix ranges from 0 to 4, representing the predicted stages from stage 0 (indicating no ROP lesions) to stage Ⅳ.

Our system for classifying the stage of ROP significantly outperformed individual ophthalmologists in both the accuracy and kappa of diagnostic predictions. In comparative testing, our model achieved an accuracy of 0.69 and a kappa score of 0.62, surpassing all five practicing ophthalmologists involved in the study, whose scores ranged from 0.37 to 0.57 for accuracy and 0.28 to 0.52 for kappa (Table 2). Additionally, our system attained higher recall than ophthalmologists for most of ROP stages, only misclassifying the most severe, stage 4 cases as the adjacent stage 3 (Figure 5).

**Figure 6:**
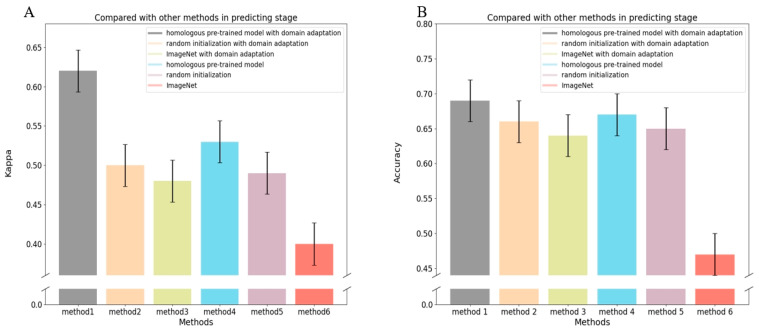
Performance of various methods in assessing the stage of ROP tasks; (**A**) represents the comparison between various methods on the kappa index in assessing the stage of ROP; (**B**) represents the comparison between various methods on the accuracy index in assessing the stage of ROP. Method 1 represents our method; method 2 represents random initialization plus domain adaptation; method 3 represents using ImageNet plus domain adaptation; method 4 represents using homologous pretrain; method 5 represents using random initialization; method 6 represents using ImageNet.

We evaluated three different model training strategies: homogeneous pre-training on retinal image datasets, random initialization, and transfer learning from ImageNet. Homogeneous pre-training on relevant retinal images achieved superior performance compared to the other approaches, indicating the benefit of building feature representations on related ROP data. Further significant improvements were attained by adding domain adaptation techniques to any of the training strategies. However, the combination of homogeneous pre-training followed by domain adaptation yielded the highest model performance for the accurate automated classifying stage of ROP (Figure 6).

### 3.2. Evaluation of the Performance for Classifying the Zone of ROP

**Figure 7:**
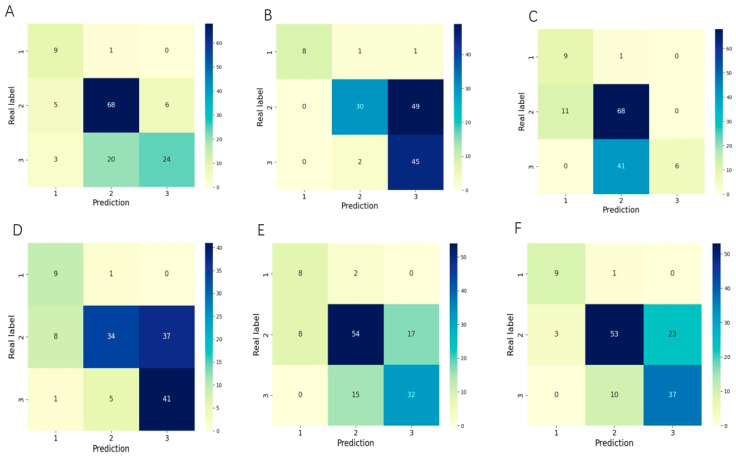
The confusion matrices of our method and clinical doctors in assessing the zone of ROP tasks: (**A**) represents our system; (**B**) represents clinical doctor A; (**C**) represents clinical doctor B; (**D**) represents clinical doctor X; (**E**) represents clinical doctor Y; (**F**) represents clinical doctor Z.

Our system also showed strong performance for classifying the zone of ROP, exceeding individual ophthalmologists in accuracy and kappa. Our model achieved an accuracy of 0.74 and a kappa of 0.55 for predicting the zone of ROP (Table 3). This accuracy surpassed all five ophthalmologists tested, who scored between 0.61 and 0.73. Our system’s kappa exceeded the majority of doctors, who ranged from 0.42 to 0.64. Additionally, our model demonstrated higher recall than ophthalmologists for most of ROP zones, while some zone III cases were conservatively misclassified as the more severe zone II (Figure 7). This direction of misclassification aligns with the clinical priority of minimizing missed cases during ROP screening, even if it increases disease over-diagnosis.

**Figure 8:**
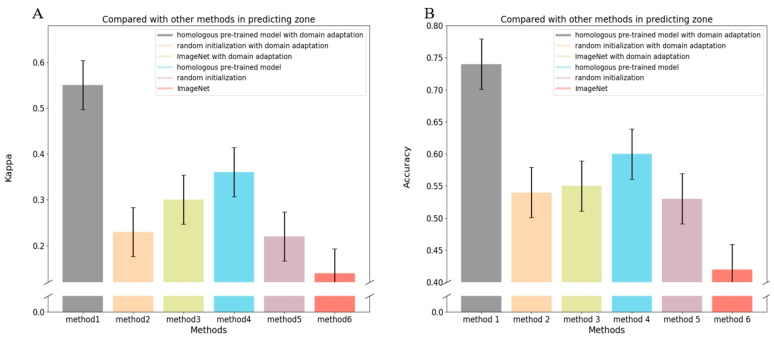
Performance of various methods in assessing the zone of ROP tasks: (**A**) represents the comparison between various methods on the kappa index in assessing the zone of ROP; (**B**) represents the comparison between various methods on the accuracy index in assessing the zone of ROP.

We also compared the three model training approaches for predicting the zone of ROP (Figure 8). Similarly, homogeneous pre-training yielded superior performance to other methods. Further sizable improvements were attained by incorporating domain adaptation into any training strategy, while the combination of pre-training on retinal images followed by domain adaptation achieved the highest model performance.

### 3.3. Evaluation of the Performance of the ROP Plus Disease Prediction

Our system again outperformed individual ophthalmologists in predicting the presence of ROP plus disease. Our model achieved an accuracy of 0.96 and an F1 score of 0.7, surpassing the doctors’ accuracy range of 0.90–0.94 and F1 range of 0.52–0.67 (Table 4). We also evaluated the impact of domain adaptation on plus disease prediction, finding that models trained with domain adaptation outperformed those without (Table 5).

### 3.4. Evaluation of the Performance of the Severity of ROP

**Figure 9:**
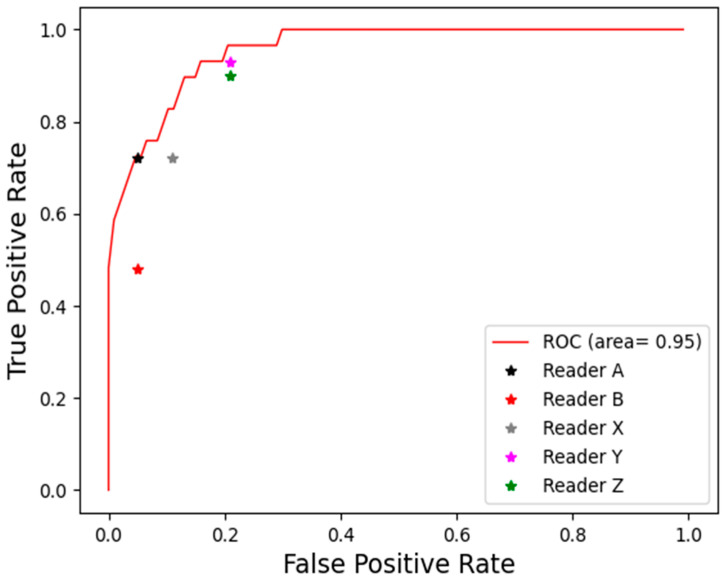
Performance in assessing the severity level of ROP tasks between our system and ophthalmologists.

Our system showed superior capabilities for evaluating overall ROP severity compared to individual ophthalmologists. Our system achieved an area under the ROC curve (AUC) of 0.95, with the ROC envelope encapsulating all doctors’ operating points (Figure 9). Additionally, as detailed in Supplemental Table S1, our system attained an accuracy of 0.91 and F1 score of 0.76 for ROP severity assessment. This matched or exceeded the performance of all five practicing ophthalmologists tested, who had accuracies of 0.81–0.90 and F1 scores of 0.58–0.76. By matching or surpassing human experts in both discrimination ability (ROC analysis) and precision/recall metrics, our machine learning approach demonstrates reliable integrated severity analysis.

**Figure 10:**
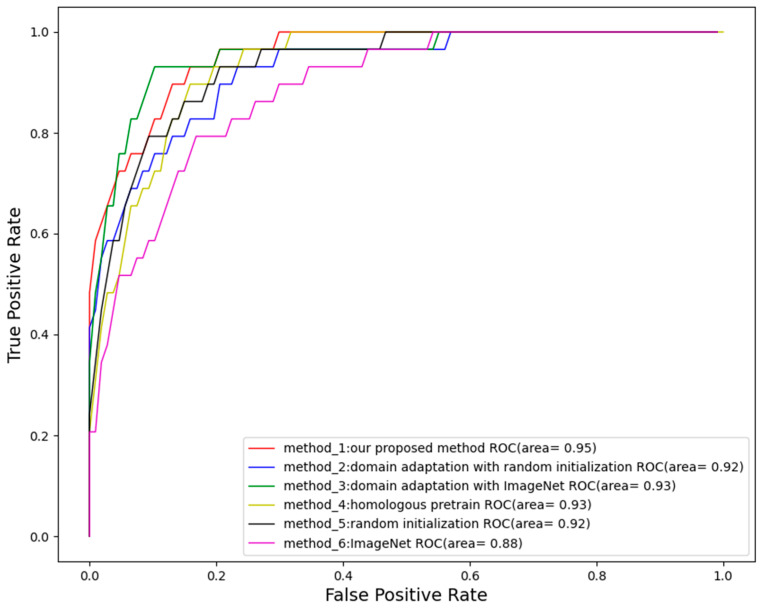
The performance of severity level of ROP between three methods which were adopted by domain adaptation: the red line represents method 1, which is our method; the blue line represents method 2, which is using domain adaptation with random initialization; the green line represents method 3, which is using domain adaptation with ImageNet; the yellow line represents method 4, which is using homologous pretrain; the black line represents method 5, which is using random initialization; the purple line represents method 6, which is using ImageNet.

With recall fixed at 1 to ensure all severe cases are identified, our method achieved much higher specificity (up to 0.7) than other methods (Table 6). This indicates that our approach maintains a lower false positive rate in detecting non-severe ROP cases, an important capability for severity screening to minimize unnecessary treatments. Our method also achieved an area under the ROC curve (AUC) of 0.95, surpassing all other methods on the AUC index in the evaluation of the performance of the severity of the ROP task (Figure 10).

### 3.5. Visualization of Our Method

**Figure 11:**
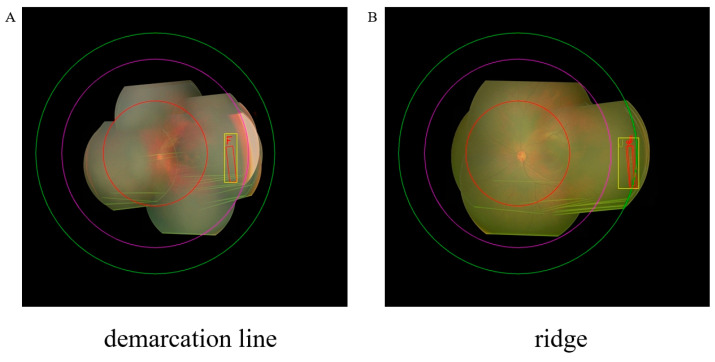

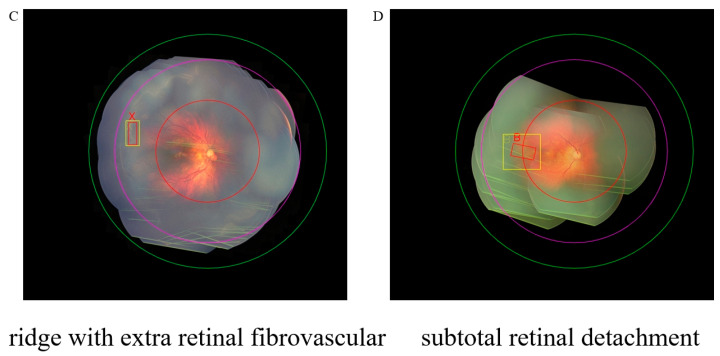
The visualization of our method. Box outlines in (**A**–**D**) indicate the type and sites of lesions: (**A**) stage I: demarcation line; (**B**) stage II: ridge; (**C**) stage III: ridge with extra retinal fibrovascular involvement; (**D**) stage IV: subtotal retinal detachment. The red circle in the middle represents zone one; the region between the purple and red circles represents zone two; and the area between the green and purple circles represents zone three. The yellow rectangle and red rectangle in the figure represent the area predicted by the model for the lesion and annotated by the doctor, respectively. The yellow letters and red letters represent the lesion type predicted and annotated by the doctor, respectively.

The visualization of our system is shown in Figure 11. When the system assesses patients, it automatically generates a panoramic view along with the final results. This view includes the optic disc, macula, and the stage and zone information of lesions. Ophthalmologists can evaluate promptly whether the system’s diagnosis is correct by browsing the information on the panoramic image and explain to the patients what causes the severity of the condition. This not only further reduces misdiagnosis but also facilitates effective communication between ophthalmologists and patients.

## 4. Discussion

Unlike previous AI studies that classify ROP severity directly from fundus images in a black-box manner [11,12,13,14,15,16,17,18,19,20,21,22,23,24,25,26], our model uniquely aligns with clinical practice guidelines by replicating the structured diagnostic process step-by-step. By first detecting the underlying criteria of the stage of ROP, the zone of ROP and plus disease presence before determining an integrated severity level, our approach provides interpretability and mimics the sequential assessments of ophthalmologists. This allows ophthalmologists to interactively review grading details, utilizing the interpretable results obtained to promptly assess the accuracy of the system’s predictions regarding lesion staging and zoning, further pinpointing problematic data, thereby reducing misdiagnosis or underdiagnosis risk, and establishing trust through enhanced transparency. Rather than acting as just a second opinion, our method serves as an interactive assistive tool that adheres to established protocols. By advancing both accuracy and process alignment with clinical practice, our work represents an important step toward safe integration of AI in ROP screening workflows, facilitating doctor–model collaboration through mutually interpretable and structured severity evaluations that can improve clinical adoption, efficiency, consistency, and ultimately patient outcomes.

Our model outperformed individual ophthalmologists in recognizing the stage of ROP, achieving an accuracy of 0.69 and kappa of 0.62. For the recognition of zone, our model attained an accuracy of 0.74, surpassing all ophthalmologists, and a kappa of 0.55, exceeding most ophthalmologists. Our system also showed superior plus disease of ROP identification to ophthalmologists, with an accuracy of 0.96 and an F1 score of 0.7. Following clinical guidelines, we integrated the recognition of stage, zone, and plus disease to determine overall ROP severity, with visual explanations of the grading. Our method matched or exceeded individual ophthalmologists in ROP severity evaluation. By accurately replicating the structured diagnostic processes of ophthalmologists, our interpretable AI system demonstrates promising capabilities to serve as an assistive tool for automated ROP assessment.

### Limitations of This Study

Our model demonstrates some limitations in identifying advanced ROP disease stages and zones, which can be attributed to imbalanced training data. As shown in Figure 5 and Figure 7, performance was lower for stage IV versus earlier stages, and some zone III cases were misclassified as zone II, reflecting the smaller sample sizes for these categories. By collecting more diverse ROP data in the future, especially for late-stage and peripheral zone III disease, we can re-balance the training set and further improve model performance. Expanding beyond our current RetCam III dataset to include other modalities like OCTA and fluorescence imaging is another valuable direction, even if their utility for ROP assessment remains limited presently. Although challenging, building a larger multi-modality ROP training corpus could strengthen our model’s capabilities across all disease stages and zones. While our current results surpass those of most individual ophthalmologists, enhancing performance on less-prevalent cases will be an important focus going forward to ensure reliable identification of the most severe and advanced disease. Currently, we can only interpret the staging and zoning information for each eye; we cannot interpret aspects related to plus disease. Our next goal is to develop a model that can interpret both staging, zoning, and plus disease.

## 5. Conclusions

In conclusion, we have developed an interpretable AI system for the automated assessment of retinopathy of prematurity (ROP) severity. Our results demonstrate that this model can accurately detect ROP stage, zone, and plus disease presence directly from fundus images. The full evaluation process is conducted automatically without human intervention, yet it also allows for interactive visualization and verification of diagnosis details. By replicating the structured diagnostic approach of ophthalmologists in a transparent manner, our system acts as an assistive screening tool that aligns with clinical guidelines. The strong performance achieved thus far highlights the potential of our AI solution to aid clinicians in ROP severity evaluation, improving efficiency, consistency, and timeliness of assessments to guide treatment decisions. In the future, we will continue to gather more data to validate and enhance our model. We remain dedicated to advancing in this field.

## Figures and Tables

**Table 1:** The information about training data and validation data for PY.

Data	Stage I Lesion	Stage II Lesion	Stage III Lesion	Stage IV Lesion
training data	77	177	39	36
validation data	11	35	10	10

**Table 2:** Compared our method with clinical doctors in assessing the performance of the stage of ROP task.

Methods	Acc	Kappa
our system	0.69	0.62
clinical doctor A	0.57	0.52
clinical doctor B	0.37	0.28
clinical doctor X	0.47	0.47
clinical doctor Y	0.51	0.45
clinical doctor Z	0.45	0.36

**Table 3:** Comparison of our method with clinical doctors in assessing the performance of the zone of ROP.

Methods	Acc	Kappa
our system	0.74	0.55
clinical doctor A	0.61	0.51
clinical doctor B	0.61	0.42
clinical doctor X	0.62	0.54
clinical doctor Y	0.68	0.59
clinical doctor Z	0.73	0.64

**Table 4:** Comparison of methods with domain adaptation techniques and clinical doctors in assessing the performance of detecting plus disease in ROP.

Methods	Acc	F1
our system	0.96	0.7
clinical doctor A	0.92	0.52
clinical doctor B	0.93	0.64
clinical doctor X	0.91	0.65
clinical doctor Y	0.94	0.67
clinical doctor Z	0.9	0.58

**Table 5:** Comparison using method and domain adaptation with clinical doctors in assessing the performance of plus of ROP task.

Methods	Acc	F1
I-ROP ASSIST with domain adaptation	0.96	0.7
I-ROP ASSIST	0.92	0.35

**Table 6:** Performance of our method and compared methods in assessing the severity level of ROP.

Methods	AUC (95%CI)	Recall	Specificity
domain adaptation with homologous pretrain	0.95 (0.90–0.98)	1	0.7
domain adaptation with random initialization	0.92 (0.86–0.96)	1	0.43
domain adaptation with ImageNet	0.93 (0.88–0.98)	1	0.45
homologous pretrain	0.93 (0.88–0.98)	1	0.68
random initialization	0.92 (0.87–0.97)	1	0.54
ImageNet	0.88 (0.81–0.94)	1	0.46

## Data Availability

The data that support the findings of this study are available from the corresponding author upon reasonable request.

## Supplementary Materials

The following supporting information can be downloaded at: https://www.mdpi.com/article/10.3390/bioengineering11080792/s1, Table S1: Performance of our method and ophthalmologists in assessing the severity level.
