# Supplementary material for: An Interpretable System for Screening the Severity Level of Retinopathy in Premature Infants Using Deep Learning
Source: Bioengineering (Basel). 2024 Aug 5;11(8):792. doi: 10.3390/bioengineering11080792 (PMC11351924; doi:10.3390/bioengineering11080792)
Supplement: Supplementary file 1 [file bioengineering-11-00792-s001.zip › bioengineering-3095174-supplementary.pdf]

Supplemental Table S1. Performance of our method and ophthalmologists in assessing the severity

methods	accuracy	F1
our system	0.91	0.76
clinical doctor A	0.9	0.76
clinical doctor B	0.85	0.58
clinical doctor X	0.85	0.68
clinical doctor Y	0.82	0.69
clinical doctor Z	0.81	0.67
